# Cartilaginous bending spring tympanoplasty: a temporal bone study and first clinical results

**DOI:** 10.1007/s00405-022-07356-0

**Published:** 2022-04-01

**Authors:** Robin Rupp, Tony Schelhorn, Stefan Kniesburges, Matthias Balk, Moritz Allner, Konstantinos Mantsopoulos, Heinrich Iro, Joachim Hornung, Antoniu-Oreste Gostian

**Affiliations:** 1 Medical Faculty, Department of Otorhinolaryngology, Head and Neck Surgery, Friedrich-Alexander-University Erlangen-Nürnberg (FAU), Waldstraße 1, 91054 Erlangen, Germany; 2 Department of Phoniatrics and Pediatric Audiology, Friedrich-Alexander-University Erlangen-Nürnberg (FAU), Erlangen, Germany

**Keywords:** Tympanoplasty, Chronic otitis media, Anterior tympanic membrane defect, Laser Doppler vibrometry, Middle ear transfer function

## Abstract

**Objective:**

Anchoring grafts for tympanic membrane (TM) reconstruction in anterior and subtotal TM defects is essential to prevent medialisation and can be facilitated by cartilaginous bending spring tympanoplasty (CBST). The purpose of this study was to analyse the impact of spring cartilages on middle ear transfer functions and patient hearing levels.

**Methods:**

In six fresh-frozen human temporal bones a cartilage graft (measuring 6 × 2 mm with a thickness of 0.1–0.2 mm) was formed into a ‘U’-shaped bending spring, to be placed between the medial tympanic wall and the tympanic underlay grafts. The stapes velocity for excitation by exponential sweeps from 400 to 10,000 Hz was measured with a laser Doppler vibrometer. The resulting middle ear transfer functions were compared with the reconstructed middle ear. For clinical evaluation, 23 ears in 21 patients with chronic otitis media and an intact ossicular chain were operated using CBST. At each follow-up visit, the patients underwent pure-tone audiometry and the Freiburg monosyllabic speech test at a presentation level of 65 dB SPL for the word recognition score (WRS).

**Results:**

The measured stapes velocities at one-third octave midband frequencies averaged 3.56 × 10^–2^ ± 9.46 × 10^–3^ (mm/s/Pa) compared to 3.06 × 10^–2^ ± 6.86 × 10^–3^ (mm/s/Pa) with the bending and underlay cartilage in place (*p* = 0.319; *r* = 0.32). The bending spring tympanoplasty reduced the transfer function by 1.41 ± 0.98 dB on average. In the clinical part of the study, the graft success rate was 96% (22 out of 23 patients) after a mean follow-up of 5.8 ± 2.4 months (min. 3.5 months, max. 12.0 months). The air–bone gap improved significantly by 6.2 dB (± 6.6 dB; *p* < 0.001; *r* = 0.69), as well as the WRS from 61.8 ± 33.3% preoperatively to 80.0 ± 20.9% postoperatively (*p* = 0.031; *r* = 0.35).

**Conclusion:**

Experimental data as well as initial clinical results suggest that CBST is an effective method for reconstructing anterior or subtotal defects of the tympanic membrane with satisfactory audiologic results and graft success rates comparable to previously described methods. It can, therefore, be added to the arsenal of tympanoplasty techniques for anterior and subtotal TM perforations.

## Introduction

Reconstructing the tympanic membrane (TM) in chronic otitis media generally yields high closure rates with satisfactory audiological outcomes [1]. Nevertheless, anterior perforations are considered more challenging, with a 67% success rate compared to 90% in posterior and inferior perforations [2]. These differences are explained by inadequate exposure, limited residual TM and poorer vascularisation of the anterior part of the TM [3, 4].

A prerequisite for successful closure of anterior and subtotal TM perforations is the secure anchorage of the graft material anteriorly. A variety of techniques use temporalis fascia as the reconstruction material. Schraff et al. described the “window shade” tympanoplasty as a combination of underlay and overlay tympanoplasty for anterior marginal TM perforations [3]. Mediolateral graft tympanoplasty uses temporalis fascia as an underlay graft at the posterior half of the perforation and as an overlay at the anterior, de-epithelialised half of the TM perforation [5]. In the mucosal pocket myringoplasty presented by Faramarzi et al., the anterior tip of the fascia graft is attached to the lateral wall of the eustachian tube orifice via a mucosal pocket [6]. Furthermore, the anterior tab flap technique uses a skin tunnel anterior to the fibrous annulus for fixating a fascia graft and was evaluated for anterior and subtotal TM perforations [7, 8].

In addition to the temporalis fascia, tragal or conchal cartilage has proved its merit in reconstructing TM perforations [9]. Especially in cases with tubal dysfunction or revision surgery, the use of cartilage can be beneficial as it better resists retraction due to its higher rigidity compared to the fascia or perichondrium [10].

Recently, the cartilaginous bending spring tympanoplasty (CBST) for anterior or subtotal tympanic membrane perforations proposed the use of a cartilage strut graft placed between the reconstructed TM and the medial tympanic wall forming a U-shaped bending spring to prevent medialization of the cartilaginous TM graft. This technique invented by Joachim Hornung was first published by Mantsopoulos et al. [11]. However, audiological properties and success rates of this new method have not yet been addressed. To validate this new technique as requested by Tos [12], we conducted an experimental temporal bone (TB) study to assess the acoustic effects of CBST alongside with a prospective clinical study.

## Materials and methods

This study was conducted at a tertiary referral medical centre (Department of Otorhinolaryngology and Head and Neck Surgery, Department of Phoniatrics and Pediatric Audiology, Friedrich-Alexander-University Erlangen-Nürnberg (FAU), Erlangen, Germany). The study was approved by the local ethics committee (application numbers: 51_18 B; 288_19 Bc).

### Temporal bone preparation

The experimental study was performed on six fresh-frozen human TBs. The TBs were collected and frozen within 24 h after death and kept at − 20 °C until they were used for the experiments within the following 3 months. A thorough microscopic inspection excluded any middle ear disease in every TB. Access to the stapes was achieved by posterior tympanotomy. Great care was taken not to harm the ossicular chain. Drilling was performed under constant rinsing with 0.9% saline solution that was also used to moisten each specimen with a spray bottle throughout the experiments every 5–10 min. A rectangular piece of reflective foil (A-RET-T010, Polytec, Waldbronn, Germany) was placed on the posterior crus of the stapes to improve the reflected vibrometer signal. In the external ear canal (EAC), a tympanomeatal flap of the posterior wall and the intact tympanic membrane (TM) were elevated to gain access to the middle ear space. A conchal cartilage graft was harvested from the same auricle and trimmed with a cartilage knife (KURZ Precise Cartilage Knife, BESS Medizintechnik GmbH, Berlin, Germany) to a thickness of about 0.3 mm before cutting it into a rectangular shape of around 4 × 4 mm with a No. 15 scalpel. For better visualization, the cartilage was stained blue with a surgical pin. This cartilage, referred to in the following text as the “underlay cartilage”, was then placed in an underlay fashion under the anterior part of the TM. Great care was taken not to touch the bony rim of the middle ear.

Thereafter, cartilage for the bending spring was trimmed with the cartilage knife to a thickness of 0.1–0.2 mm and cut into a rectangular shape of 6 × 2 mm. The cartilaginous bending spring, in the following referred to as the “spring cartilage”, was then placed in the hypotympanum between the medial tympanic wall and the underlay cartilage of the anterior TM forming an anteriorly orientated U-shape. Subsequently, the TM together with the tympanomeatal flap were laid back.

### Test sequence

All experiments were conducted in the same test sequence order for each TB. At first, measurements of the stapes velocity at the posterior crus were carried out prior to any manipulation of the TM. Only TBs comparable to the requirements described by Rosowski et al. were used for further measurements [13]. Then, the baseline measurement was carried out after elevating the tympanomeatal flap and the TM. Subsequently, the underlay and spring cartilages were placed accordingly. Finally, after removal of both grafts, the baseline measurements were repeated.

### Temporal bone measurements

The transfer functions were acquired by measuring the posterior crus velocities related to the sound pressure of the excitation signal applied in the EAC.

The EAC was sealed with a disposable foam eartip (ER1-14B, Etymotic Research, Elk Grove Village, IL, USA) that contained the tubing of the sound source (ER-2, Etymotic Research, Elk Grove Village, IL, USA) producing the excitation signal and the reference microphone (ER-7C, Etymotic Research, Elk Grove Village, IL, USA) measuring the sound pressure level (SPL) at the eardrum.

The laser Doppler vibrometer (LDV) (OFV-5000, Polytec, Waldbronn, Germany) was pointed at the reflective foil placed on the posterior crus of the stapes to measure the stapes velocity. Cosine correction was not performed. Alignment of the laser beam with the reflective foil was facilitated manually by a micromanipulator (A-HLV-MM30, Polytec, Waldbronn, Germany) attached to the head of the LDV fixed on the microscope.

An exponential sweep with a sampling rate of 44.1 kHz set to approximately 93 dB (SPL) in the ear canal was used as the excitation signal. The frequency range was from 400 to 10 kHz. One sweep contained 218 samples, resulting in a sweep signal length of 5.94 s. The output signals from the microphone and LDV were received by a sound card (HDSP 9632, RME, Haimhausen, Germany) which also drove the sound source. Each measurement was repeated three times. The average of these three repetitions was used for all subsequent calculations. The measured velocities of the stapes were compared using the mean values at one-third octave midband frequencies within the frequency range.

**Fig. 1:**
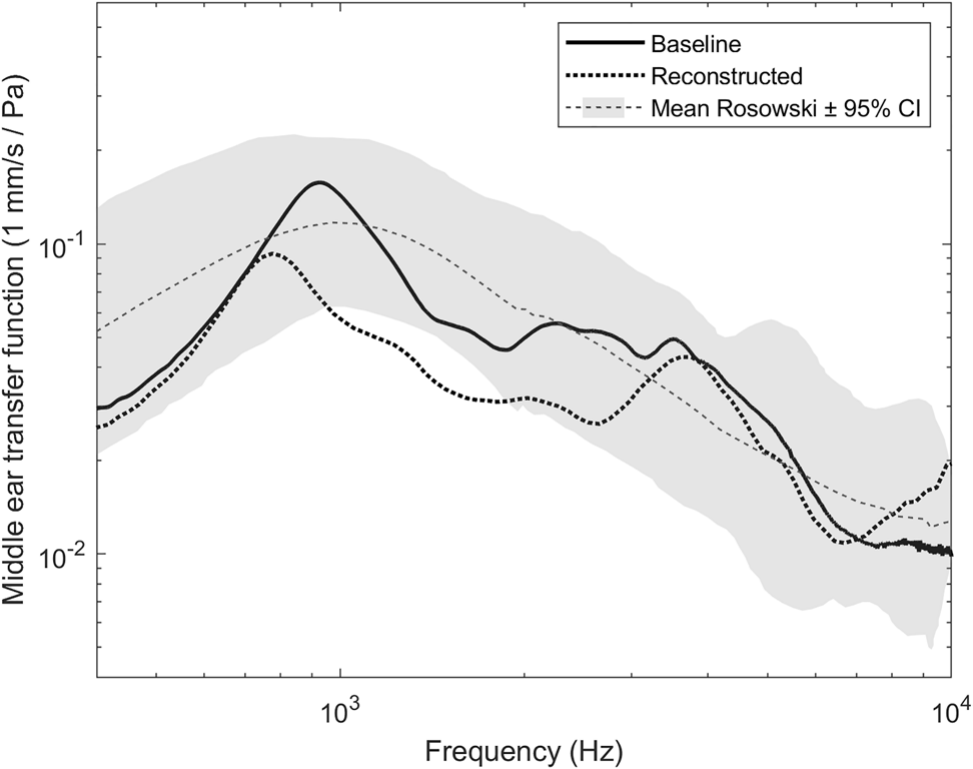
Exemplary results of the middle ear transfer functions from TB #3. Baseline = baseline measurement without reconstruction; Reconstructed = with the underlay and spring cartilage in place; Mean Rosowski CI ± 95% = confidence interval as described by Rosowski et al. [13]; *Pa* pascal, *Hz* hertz

A computer containing the sound card was used to create and analyse the signals in MATLAB using the ITA-Toolbox (RWTH Aachen University, Aachen, Germany, http://www.ita-toolbox.org). All signals were inspected in real time with an oscilloscope (TDS 2004C, Tektronix, Beaverton, USA). Additionally, the posterior crus displacement was calculated from the velocity by integrating over time to further verify the correctness of the setup. The impulse responses of the microphone and LDV were acquired by deconvolution with the excitation signal. Windowing in the time domain was performed on the impulse responses to further increase the signal-to-noise ratio (SNR). Dividing the LDV impulse responses by the reference microphone impulse responses resulted in the middle ear transfer functions that were compared between the TBs (Fig. 1).

### Clinical data and surgical technique

Clinically, prospective data collection from consecutive patients was performed between March 1st, 2018 and January 31st, 2021 and included the following inclusion criteria: tympanoplasty type I in chronic otitis media for reconstruction of a defect of the TM that affected at least the anterior part of the TM including subtotal defects; intact ossicular chain, age ≥ 18 years. The following exclusion criteria applied: evidence of cholesteatoma; reconstruction of the ossicular chain; TM defects not affecting the anterior part of the TM, lack of follow-up, refusal to participate. All patients included gave their written informed consent to participate. Prior to the tympanoplasty and at each follow-up visit, each patient underwent pure-tone audiometry and the Freiburg monosyllabic speech test at a presentation level of 65 dB SPL for the word recognition score (WRS). Follow-up for audiometry was scheduled 3, 6 and 12 months postoperatively. Hearing results were presented by the mean of the four-frequency pure-tone average (4FPTA), i.e. 0.5, 1, 2, and 3 kHz and the WRS.

**Fig. 2:**
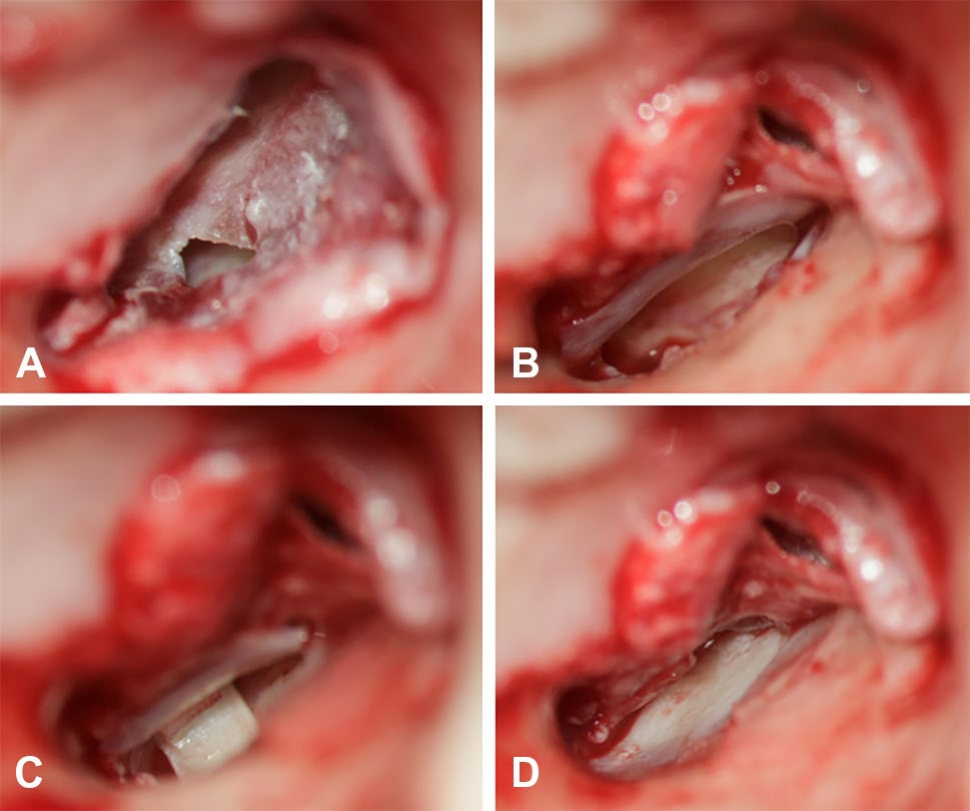
Intraoperative documentation of essential steps of cartilaginous bending spring tympanoplasty. **A** Patient with chronic otitis media showing a defect in the anterior inferior part of the tympanic membrane. **B** A cartilage plate is placed as an underlay graft. **C** A U-shaped bending spring cartilage is placed between the medial bony wall of the tympanic cavity and the underlay cartilage. **D** Final position of the underlay cartilage covering the tympanic membrane defect

All tympanoplasties were performed by two experienced otologic surgeons (JH and AOG) in general anaesthesia. A retroauricular or transmeatal approach was used at the surgeon’s discretion. A detailed description of the surgical procedure of cartilaginous bending spring tympanoplasty is given by Mantsopoulos et al. [11]. In brief, surgery was performed under general anaesthesia using an endaural or retroauricular incision. A tragal or conchal cartilage graft as well as a perichondrium graft were harvested. All cartilages were thinned with the KURZ Precise Cartilage Knife (BESS Medizintechnik GmbH, Berlin, Germany). A tympanomeatal flap was raised and the underlay cartilage, thinned to approximately 0.3 mm, was placed together with the perichondrium as an underlay graft to cover the defect of the TM. Following this, the spring cartilage was trimmed to a thickness of 0.1–0.2 mm and cut into a rectangular shape (length: 6 mm, width: 2 mm) with a No. 15 scalpel. The cartilage was then placed in the hypotympanum forming a U-shaped bending spring between the medial bony wall of the tympanic cavity and the underlay cartilage to prevent its medialisation. The GELITA-SPON^®^ STANDARD ear dressing (Gelita Medical, Eberbach, Germany) was removed 2–3 weeks later. Figure 2 shows intraoperative documentation of essential steps of CBST.

### Statistical analysis

Metric variables are presented as values ± standard deviation (SD) and range [minimum (min.), maximum (max.)]. Normal distribution was tested using the Kolmogorov–Smirnov test. Accordingly, independent *t* tests were performed to assess the measured stapes velocities and dependent *t* tests to evaluate hearing improvement after surgery. The 95% confidence interval (CI) for difference of means was calculated for parametric tests. Non-normally distributed metric variables were analysed using the Wilcoxon signed rank test. A *p* value of < 0.05 was considered as statistically significant. For *t* tests and the Wilcoxon signed rank test, *r* is presented as an effect size with 0.1 equalling a small, 0.3 a moderate and 0.5 a strong effect. Statistical analysis was performed using SPSS (IBM SPSS Statistics 24; IBM, New York, NY, USA).

## Results

### Experimental evaluation

The measured stapes velocities of the used TB at one-third octave midband frequencies averaged 3.56 × 10^–2^ ± 9.46 × 10^–3^ (mm/s/Pa) compared to 3.06 × 10^–2^ ± 6.86 × 10^–3^ (mm/s/Pa) with the bending and underlay cartilage in place (*t*(10) = 1.05; *p* = 0.319, *r* = 0.32; 95% CI − 5.63 × 10^–3^ to 1.56 × 10^–2^). Analysing low frequencies only (400–630 Hz), the stapes velocities without the bending cartilage in place measured 3.00 × 10^–2^ ± 1.14 × 10^–2^ compared to 3.11 × 10^–2^ ± 1.36 × 10^–2^ with the bending cartilage (*t*(10) = − 0.15; *p* = 0.886; *r* = 0.05; 95% CI − 1.72 × 10^–2^ to 1.50 × 10^–2^). The stapes velocities for high frequencies (800 Hz–10 kHz) amounted to 3.71 × 10^–2^ ± 1.16 × 10^–2^ and 3.05 × 10^–2^ ± 9.29 × 10^–3^ with and without the bending cartilage, respectively (*t*(10) = 1.08; *p* = 0.308, *r* = 0.32; 95% CI − 6.99 × 10^–3^ to 2.00 × 10^–2^).

**Fig. 3:**
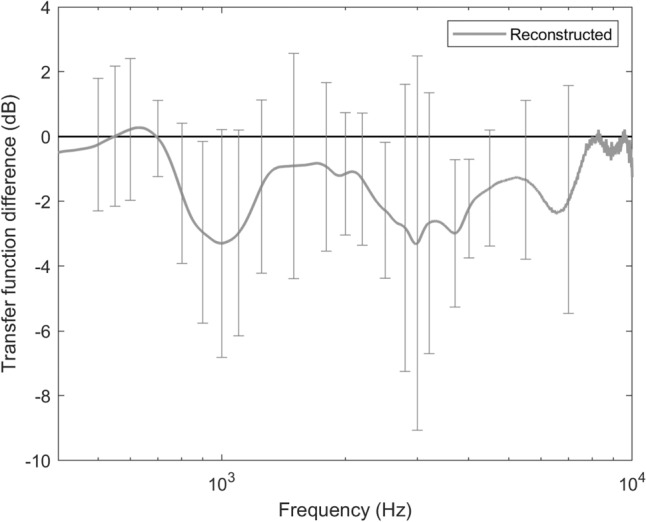
Mean differences in middle ear transfer functions with underlay and spring cartilages in place as compared to baseline measurements; Baseline measurements are depicted by the horizontal line at 0 dB. The grey line represents the mean transfer function from all temporal bones; error bars depict ± standard deviation. *dB* decibel, *Hz* hertz

CBST reduced the transfer function over the entire frequency range (400 Hz–10 kHz) by 1.41 ± 0.98 dB on average (Fig. 3). The transfer function reduction, averaged over low frequencies (400–630 Hz), amounted to 0.16 ± 1.92 dB, while the average for high frequencies (800 Hz–10 kHz) was 1.72 ± 1.23 dB.

### Clinical evaluation

The included 23 ears accounted for 21 patients (15 ♀, 10 right ears) who averaged 46.0 years (± 13.7 years, min. 23.4 years, max. 75.3 years) at the time of surgery. 15 ears (65%) showed a TM defect that involved the anterior part of the TM only, whereas 8 ears (35%) had a subtotal perforation. Four ears (17%), 1 ♀, 2 right ears; 28.0 ± 6.6 years, min. 23.6 years, max 37.8 years) had undergone one (*n* = 3) and two (*n* = 1) previous tympanoplasties.

Mean operation time was 71.8 min (± 22.0 min, min. 46 min, max. 124 min).

A graft success rate of 96% (22 out of 23 ears) was achieved after a mean follow-up of 5.8 months (± 2.4 months, min. 3.5 months, max. 12.0 months). In one case with subtotal TM perforation, the TM developed a new retraction pocket 7 months postoperatively needing revision surgery. Intraoperatively, the underlay cartilage was found to have migrated away from the spring cartilage.

**Fig. 4:**
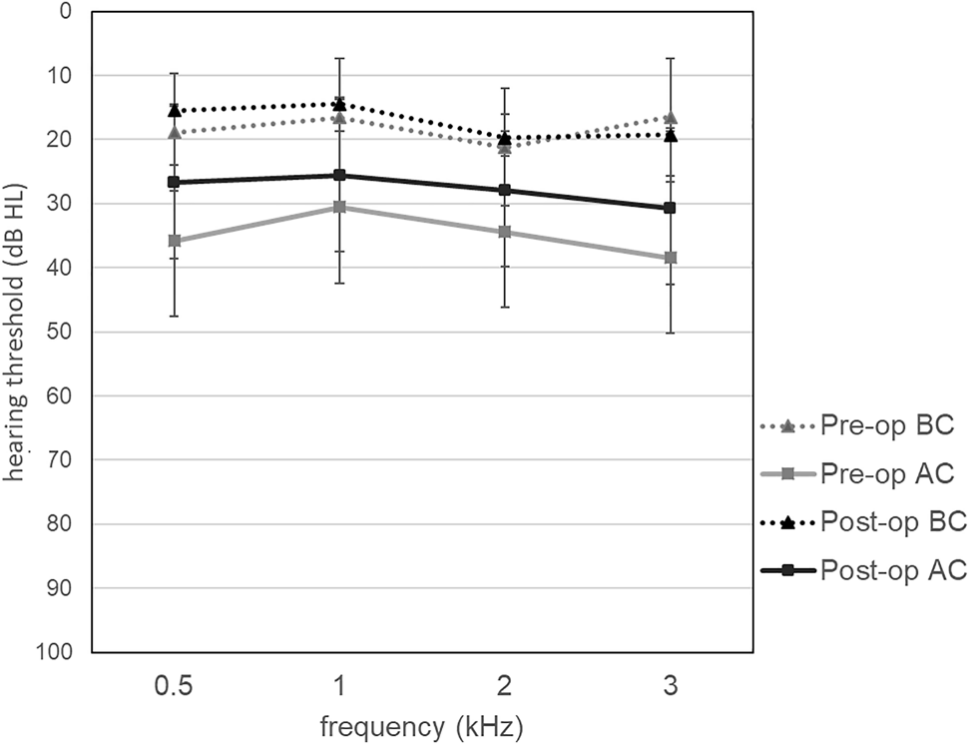
Pre- and postoperative pure-tone average thresholds; *AC* air conduction, *BC* bone conduction, *pre-op* preoperative, *post-op* postoperative, *dB HL* decibel hearing loss, *kHz* kilohertz

**Fig. 5:**
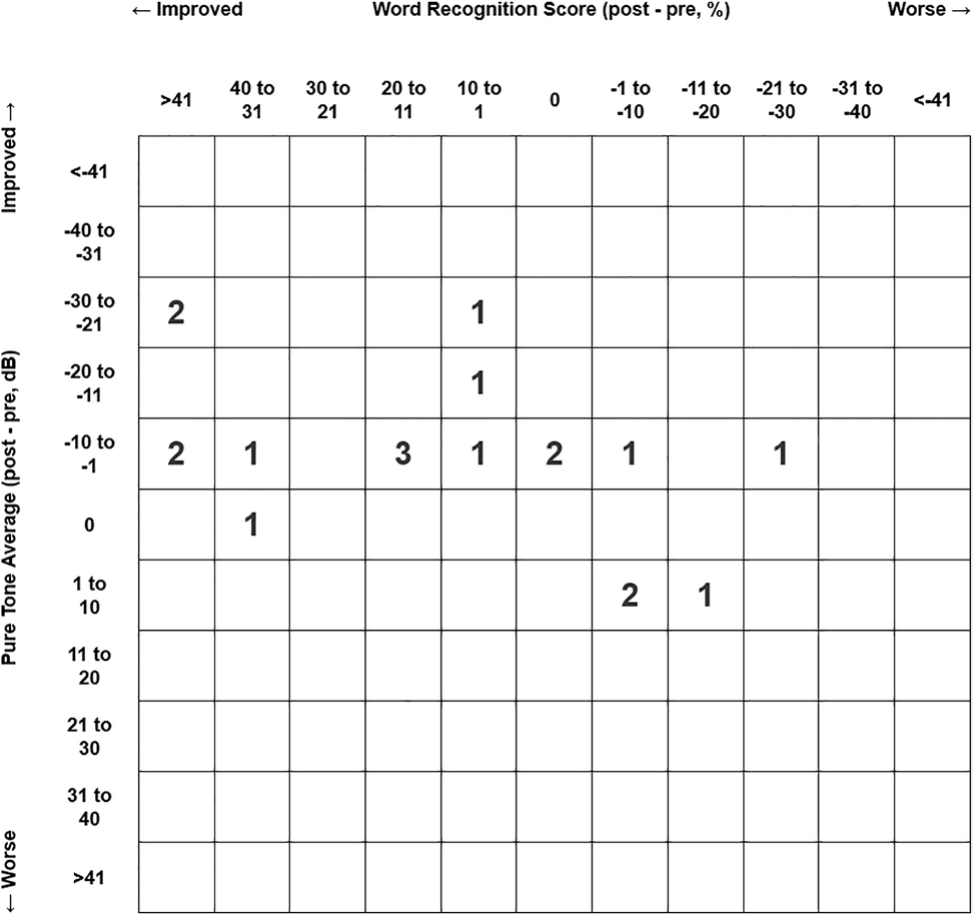
Scattergram relating changes of pre- and postoperative 4FPTA to changes in WRS; *dB* decibel

The preoperative 4FPTA for bone conduction (18.4 ± 7.2 dB; min. 4 dB; max. 30 dB) remained stable during follow-up (17.4 ± 8.9 dB; min. 1 dB; max. 38 dB; *t*(22) = 0.97; *p* = 0.342; *r* = 0.20; 95% CI − 1.18 to 3.27). The 4FPTA for air conduction improved significantly from 35.0 dB (± 9.1 dB, min. 23 dB, max 54 dB) preoperatively to 28.0 dB (± 10.7 dB; min. 2 dB; max. 46 dB; *t*(22) = 4.18; *p* < 0.001; *r* = 0.67; 95% CI 3.55 to 10.54) postoperatively. Accordingly, the mean 4FPTA air-bone gap (ABG) improved significantly from 16.7 ± 6.5 dB preoperatively to 10.6 ± 5.6 dB postoperatively by 6.2 dB (± 6.6 dB, min. − 4 dB, max. 21 dB; *t*(22) = 4.47; *p* < 0.001; *r* = 0.69; 95% CI 3.29–8.98). This resulted in postoperative ABG < 10 dB in 12 cases (52.2%), 11–20 dB in 10 cases (43.5%) and 21–30 dB in 1 case (4.3%). Likewise, WRS @ 65 dB increased significantly from 61.8 ± 33.3% (*n* = 22; min. 0%; max. 100%) preoperatively to 80.0 ± 20.9% (*n* = 19; min. 30%; max. 100%; *Z* = 2.158; *p* = 0.031; *r* = 0.35) postoperatively. Pre- and postoperative pure-tone average thresholds are shown in Fig. 4. The scattergram (Fig. 5) depicts changes of pre- and postoperative 4FPTA in relation to WRS according to Gurgel et al. [14] and was created using a web-based tool [15].

## Discussion

The experimental data presented in this study indicate that CBST does not significantly impair the acoustic transfer function of the reconstructed middle ear. Clinical results show a high graft success rate combined with favourable audiological results in short-term follow-up. Therefore, CBST can be advocated as an effective method for reconstructing anterior or subtotal TM defects.

Tympanoplasty techniques using cartilage are widely used due to the proven beneficial properties of cartilage and are specially emphasised in challenging cases mostly represented by tubal dysfunction and large or recurrent perforations due to increased stability compared to the fascia or perichondrium [10]. Yet, cartilage thickness distinctly influences the acoustic transmission of the reconstructed middle ear. Mürbe et al. demonstrated that cartilage plates with a thickness of 0.3 and 0.5 mm have favourable acoustic properties compared to those of 0.7 and 1.0 mm [16]. In a biomechanical analysis, Lee et al. described the optimal thickness of a cartilage graft for medium and large TM perforations as between 0.1 and 0.2 mm and for small perforations as less than 1.0 mm [17]. Due to the vast variety of cartilage tympanoplasty methods, Tos proposed a classification including 23 different techniques. In this context, CBST is a modification of a technique described as underlay tympanoplasty with cartilage foils and plates where cartilage grafts with a thickness of 0.2–0.4 mm are placed under the remaining eardrum [12]. In CBST, we used a cartilage plate with a thickness of 0.3 mm as the underlay cartilage supported by a bending spring cartilage.

Methods described for the closure of defects including the anterior part of the TM favour the temporalis fascia, allowing success rates of between 67 and 98.8% [2, 3, 5–8, 18, 19]. Thus, our CBST results with respect to graft success rates are comparable. With regard to hearing restoration, Faramarzi et al. reported a postoperative ABG < 25 dB in 75% of patients with a normal ossicular chain following the anterior tab flap technique in large TM perforations in a collective with a slightly higher preoperative air conduction [7]. In the present study, 95.7% of the reconstructed ears had a postoperative ABG < 20 dB that is also comparable to the 98% reported by Schraff et al. However, they found more patients, i.e. 82%, with an ABG < 10 dB compared to 52.2% in our study, which may be attributed to the fact that they included marginal anterior perforations only [3]. Lee et al. report a postoperative ABG < 20 dB in 93% of cases with an ABG < 10 dB in 80.4%, the mean follow-up being 15.2 months compared to 5.8 months in the present study [18]. A slightly higher reduction of the ABG (9.75 dB) than in the present study (6.2 dB) was reported by Harris et al., although the study comprised a smaller cohort with a higher mean preoperative ABG [8].

In their experimental study evaluating the acoustic effects of various cartilage overlays on the TM, Eldaebes et al. reported a significant correlation between the cartilage mass and losses in the stapes velocity for low and middle frequencies. The effect on acoustic transfer function was less than 10 dB even for complete cartilaginous covering of the TM [20]. These only modest modifications of transfer function are in line with the findings of the present study. Interestingly, the measured stapes velocities at high frequencies seem to be more affected by CBST, displaying a medium effect size, than at low frequencies, showing a small effect size.

Several limitations of the study have to be addressed. The foremost limitation of the clinical evaluation includes the inevitable bias caused by the study design of consecutively enrolling patients and the relatively short follow-up together with the relatively low but comparable number of evaluated ears. Additionally, tympanoplasties were performed using both the transmeatal and retroauricular approach displaying a potentially influencing factor. It is of note that the cartilage thicknesses in both the clinical and experimental evaluations were not determined exactly by pathological evaluation but estimated accurately by the use of the clinically available Precise Cartilage Knife that is routinely used during surgery. With regard to the experimental part, changes in the acoustic properties of the TB during measurements over time could not be ruled out, although experiments were carried out within a few hours the same day and the specimens were kept well moistened. Furthermore, a cosine correction was not performed when calculating the transfer functions.

The combination of both the experimental and the clinical assessment of CBST allow a preliminary comprehensive evaluation that merits further investigations on a larger number of patients with a longer follow-up to determine the full potential of CBST.

In conclusion, experimental data as well as initial clinical results suggest that CBST is an effective method for reconstructing anterior or subtotal defects of the TM with satisfactory audiologic results and graft success rates comparable to previously described methods. It can, therefore, be added to the arsenal of tympanoplasty techniques for anterior and subtotal perforations.
